# Management of Dry Eye Disease Pre- and Post-Cataract Surgery: A Personalized Approach

**DOI:** 10.3390/jpm16020086

**Published:** 2026-02-03

**Authors:** Samantha Spritz, Raul E. Ruiz-Lozano, Zahra Bibak-Bejandi, Nicholas W. Setter, Alejandro Rodriguez-Garcia, Zeenal Dabre, Ali Khodor, Robert Schwartz, Sandeep Jain, Ali R. Djalilian

**Affiliations:** 1 Department of Ophthalmology and Visual Sciences, Illinois Eye and Ear Infirmary, University of Illinois Chicago, Chicago, IL 60612, USA; sspri@uic.edu (S.S.); raule.ruiz91@gmail.com (R.E.R.-L.); zhrbibak@uic.edu (Z.B.-B.); settern@uic.edu (N.W.S.); zdabre@uic.edu (Z.D.); rschw@uic.edu (R.S.); jains@uic.edu (S.J.); 2 Institute of Ophthalmology and Visual Sciences, School of Medicine and Health Sciences, Tecnologico de Monterrey, Monterrey 66278, Mexico; arodri@tec.mx; 3 Bascom Palmer Eye Institute, University of Miami, Miami, FL 33136, USA; ali.kh.9b@gmail.com

**Keywords:** cataract, cataract surgery, cornea, dry eye disease, keratometry, ocular surface, phacoemulsification, ocular biometry, optical aberration, tear film

## Abstract

Dry eye disease (DED) is a common condition that can be associated with cataract surgery, requiring pre- and postoperative considerations. Pre-existent DED and disruption of the tear film homeostasis due to incisional corneal nerve damage, intra-operative ocular surface drying, microscope phototoxicity, or the toxic effects of preservatives and active ingredients of postoperative drops or a combination thereof, represents a potential mechanism for worsening or developing DED after cataract surgery. Recent diagnostic advancements have enabled us better to understand the pathophysiology of DED after cataract surgery. For patients with pre-existing DED before cataract surgery, early intervention can improve surgical outcomes. In contrast, failure to recognize DED risk factors or subtle signs can result in inaccurate refractive measurements, poor surgical outcomes, including serious complications, worsening of dry eye symptoms, patient dissatisfaction, and decreased quality of life. This review presents an overview of the perioperative management of DED in patients undergoing cataract surgery with an emphasis on pre-operative diagnosis and treatment, and its impact on improving surgical refractive outcomes and decreasing complications.

## 1. Introduction

Dry eye disease (DED) is a rising multifactorial symptomatic disease affecting the ocular surface through loss of tear film homeostasis and neurosensory abnormalities [1]. It is one of the common reasons for consultation in eye clinics worldwide, contributing to an estimated economic burden of $55 billion annually in the United States, $1.10 million in the United Kingdom, and $0.27 million in France [2]. Data on economic burden in China is estimated to range from $104.2 billion to $166.6 billion annually [3]. DED presents with a range of symptoms that can vary in severity and may include dryness, a sensation of a foreign body in the eye, burning, or fluctuating blurry vision, often in combination. These symptoms can significantly impact an individual’s quality of life (QoL) [4]. DED can lead to damage to the ocular surface and visual disturbances, and in severe, untreated cases, this damage may be irreversible [5].

DED is linked to a variety of risk factors, including demographic, environmental, nutritional, systemic, and ophthalmic disorders, as well as the use of certain medications [6]. Some systemic diseases that contribute to the risk of DED development after cataract surgery include thyroid malfunction, diabetes, and autoimmune disorders [7]. Prior eye surgery is another risk factor with cataract surgery being the most frequently performed ophthalmic procedure in current clinical practice [8]. While advances in cataract surgery techniques over the past few decades have resulted in excellent outcomes for many patients, some still experience post-operative complications such as DED, which can lead to patient dissatisfaction and a decreased QoL [7,9].

The pathophysiology of DED after cataract surgery remains to be fully elucidated. However, research indicates that cataract surgery can both induce and worsen pre-existing DED [9]. Furthermore, pre-existing DED may affect the accuracy of pre-operative topographic and biometric measurements, leading to intraocular lens (IOL) power miscalculation and poor post-operative visual and unexpected residual refractive outcomes [10]. Therefore, it is crucial to properly address DED both before and after cataract surgery to minimize complications and enhance overall outcomes.

This narrative review outlines the current understanding of preoperative, intraoperative, and postoperative considerations for patients with DED undergoing cataract surgery. Understanding how to optimize the ocular surface in patients with DED helps ensure accurate biometric calculations and enhance visual outcomes.

## 2. Materials and Methods

A systematized literature search was conducted on 14 September 2025 using the National Library of Medicine’s PubMed, Google Scholar, and Scopus databases for all English language articles published until September 2025. All study designs were considered for inclusion to ensure a comprehensive dataset (e.g., randomized controlled trials, cohort studies, case series, etc.). Search terms were used alone or in combination with at least one other term and were linked using the Boolean operators “AND”, “OR”, and “NOT.” Relevant references from the articles found were also included. The following search terms were used: “dry eye disease”, “DED”, “dry eye syndrome”, “ocular surface disease”, “cataract”, “cataract surgery”, “extracapsular cataract extraction”, “phacoemulsification”, “femtosecond laser-assisted cataract surgery”, “FLACS”, “intraocular lens”, “ocular biometry”, “optical aberrations”, “keratometry”, “preoperative evaluation”, “dry eye questionnaires”, “vital dyes”, “lid wiper epitheliopathy”, “meibomian gland”, “TFBUT”, “corneal nerves”, “corneal sensitivity”, “tear hyperosmolarity”, “matrix metalloproteinase”, “artificial tears”, “topical corticosteroids”, “cyclosporine A”, “lifitegrast”, “lactoferrin”, “autologous serum”, “contact lens”, “non-steroidal anti-inflammatory drugs”, and “diquafasol”. The Boolean operators “AND”, “OR”, and “NOT” were used to maximize the retrieval of relevant articles. Relevant references from the articles found were also included.

## 3. Prevalence of Dry Eye Disease in Patients Undergoing Cataract Surgery

DED is one of the most encountered eye conditions in both general and specialized eye clinics. Determining the prevalence of DED is complicated due to various factors, including geographical, environmental, demographic, and racial differences, as well as the inconsistent criteria used to diagnose DED in population-based studies [4]. According to the Tear Film & Ocular Surface Society (TFOS) Dry Eye Workshop (DEWS) II Epidemiology Report, the prevalence of DED varies significantly: it ranges from 4.9–52.9% based on symptoms, from 6.0–98.5% based on signs, and from 8.7–10.31% when both symptoms and signs are considered for diagnosis [11]. A Bayesian analysis by Papas estimated the global prevalence of DED to be 11.59%, with a linear increase noted after the age of 50 [12]. The TFOS DEWS III Executive Summary expands upon previous prevalence rates to report that not all DED subtypes follow the pattern of higher prevalence in females and older individuals; however, studies that follow the Women’s Health Study criteria do follow this pattern [13].

A systematic review and meta-analysis from 2022 indicated that 37.4% (95%-confidence interval [CI]: 22.6–52.3) of patients who underwent cataract surgery developed DED [7]. The primary risk factors for developing post-cataract surgery DED included age, female sex, lifestyle habits, pre-existing DED, systemic diseases and medications, psychiatric conditions, meibomian gland dysfunction (MGD), the use of preserved eyedrops, and specific surgical techniques [7].

## 4. An Overview of the Pathophysiology of Dry Eye Disease

TFOS-DEWS II and III consistently define DED as a multifactorial condition that may be characterized by the loss of homeostasis in the ocular surface or the tear film. This disruption leads to tear film instability, hyperosmolarity, ocular surface inflammation, and neurosensory abnormalities [1]. An instability or imbalance of the tear film may be reflected in a shorter tear film breakup time (TFBUT), or a decreased tear meniscus height, although this is not explicitly necessary for a diagnosis of DED [14]. This instability can be caused by decreased tear production, altered composition, or delayed clearance [15,16]. If tear hyperosmolarity results, it may trigger an inflammatory response in the ocular surface, causing epithelial damage, apoptosis of conjunctival goblet cells, reduced production of mucins, and ultimately, an unstable tear film [16,17,18,19]. Tear film instability and hyperosmolarity can occur due to either decreased tear production (aqueous-deficient DED), increased evaporation (evaporative DED), or a combination of both (mixed type). This negative vicious cycle forms the core pathophysiological mechanism that perpetuates DED [15]. Overall, the reduction in tear wetting combined with increased inflammation on the ocular surface can potentially affect topographic and biometric assessments in patients undergoing cataract surgery [10].

## 5. The Influence of Dry Eye Disease in Ocular Biometry for Cataract Surgery

There are several key factors that contribute to achieving optimal visual outcomes in cataract surgery. These include a thorough diagnosis and proper preparation of the patient, performing a clean surgery with minimal complications or effectively managing any complications that arise [20]. Additionally, accurate calculation and selection of the IOL are crucial. Ultimately, maintaining a healthy ocular surface is essential for achieving good visual quality. A stable tear film is the most critical refractive surface of the eye, which contributes to high vision quality and quantity. This, in turn, ensures patient satisfaction and fosters better personal relationships [21].

### 5.1. Optical Aberrations in Patients with Dry Eye Disease

The impact of a dysfunctional ocular surface and an unstable tear film on visual outcomes after cataract surgery is primarily observed in the calculation and selection of IOLs. This effect arises from the instability in corneal measurements, alterations in topographic images, and difficulties in accurately determining the appropriate IOL [22].

The tear film is the most anterior refractive surface of the eye, playing a crucial optical role in maintaining the quality of vision (QoV) [23]. Its properties include a thickness that ranges from 6 to 20 µm when the eye is open, a volume of 5 to 10 µL, and an evaporation rate of 9.5 × 1^−7^ g/s. The refractive index of the tear film is 1.337, which when combined with the cornea, yields a refractive power of 42.36 diopters (D) [24]. If there is a uniform reduction of the tear film thickness, it will have a minimal effect on aberrations or surface power, as the surface radius can change by a maximum of 20 mm. This change results in a maximum power increase of about 0.10 D. However, when the tear film’s thickness is decreased or becomes irregular, as seen in patients with DED or those with irregular blinking patterns, significantly larger variations occur. These variations are known as high-order aberrations (HOAs) [24,25,26]. Consequently, any irregularity in tear film can lead to optical aberrations and a decrease in the QoV.

**Figure 1:**
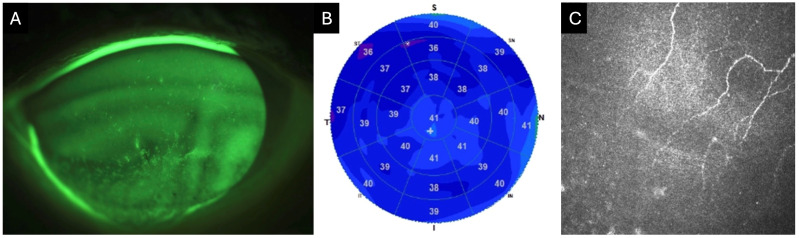
Clinical images of an 82-year-old female with neurotrophic keratitis (Cochet–Bonnet corneal sensitivity: 20 mm). (**A**) Corneal fluorescein staining demonstrates superficial punctate keratitis inferiorly (3 points), temporally (1 point), nasally (1 point), and centrally (1 point), for a total National Eye Institute (NEI) grading score of 6. (**B**) Corneal epithelial optical coherence tomography (OCT) map shows diffuse epithelial thinning, as represented by the extensive blue zone. Cool-toned colors (i.e., dark blue) repesent thinner areas with warm colors (i.e., red and orange) representing thicker areas of epithelium. (**C**) In vivo confocal microscopy (IVCM) at the level of the sub-basal nerve plexus reveals markedly reduced corneal nerves.

Non-invasive techniques for measuring the optical quality of the air-tear film interface include the double-pass method, videokeratoscopy, retroillumination, wavefront aberrometry, and interferometry. Detailed description of each of these processes can be found in other sources [26]. Tear film instability leads to increased HOAs and ocular light scattering, which results in vision fluctuations during blinking and glare, respectively [27]. Koh et al. analyzed the sequential changes in HOAs after blinking in patients with DED both with and without central superficial punctate keratopathy (SPK) using a wavefront sensor [28]. They found that DED patients with central SPK exhibited significantly higher coma-like, spherical-like, and total HOAs compared with those without central SPK. Sequential post-blink measurements were consistently higher in eyes with DED and central SPK. In contrast, eyes without central SPK demonstrated consistently lower total HOAs that resembled the patterns found in healthy eyes [28]. Therefore, it is crucial to consider not only a diagnosis of DED but also the presence of irregular epithelial surface in the optical zone (Figure 1).

Patients with DED associated with systemic inflammatory diseases deserve special attention, as these individuals often experience a severely compromised ocular surface [29]. Chronic graft-versus-host disease (GvHD) is a serious complication that can occur following hematopoietic stem cell transplantation (HSCT) for hematologic cancers [30,31,32]. DED is the most prevalent manifestation of chronic GvHD involving the eyes [33]. Studies have consistently reported higher rates of complications, such as corneal epithelial defects, corneal melts, and filamentary keratitis, in these patients following cataract surgery [34,35,36]. Our group demonstrated that with appropriate preoperative, intraoperative, and postoperative care, the complication rate can be significantly reduced [37]. The results confirm that meticulous perioperative management of dry eyes can significantly improve surgical outcomes. Research by Shimizu et al. has shown that patients with ocular GvHD exhibit significantly higher total and anterior HOAs and spherical aberration at both 4- and 6-mm diameters compared to healthy controls [38]. In contrast, Sjögren syndrome (SS) is a chronic inflammatory disease affecting multiple systems, including the eyes [39]. Autoantibodies and lymphocytes play a role in causing inflammatory dry eye symptoms in patients with SS, and the severity of DED in these patients differs from those with non-SS DED [40]. A study by Denoyer et al. reported that both SS-associated and non-SS-associated DED demonstrated significantly higher total HOAs, as well as coma and trefoil aberrations compared to controls. Additionally, there were noted negative and positive correlations between the progression of HOAs and scores from the TFBUT and Ocular Surface Disease Index (OSDI), respectively [40].

### 5.2. Variations in Keratometry in Dry Eye Disease

Accurate keratometry is essential for precise IOL calculation. Keratometers measure corneal curvature by analyzing the reflected light from the cornea. Consequently, any distortion of the tear film can lead to inaccurate measurements. In a recent study involving patients undergoing cataract surgery, Yang et al. reported a statistically significant difference in the mean keratometry (ΔKm; 0.09 D vs. 0.28 D, *p* = 0.005) and astigmatic vector (ΔKvector; 0.22 D vs. 0.50 D, *p* = 0.010) between eyes with DED eyes and healthy controls measured with the OA-2000 (Tomey Corporation, Nagoya, Japan). Additionally, the percentage of eyes with ΔKm and ΔKvector values exceeding 0.5 D was significantly different between the two groups (*p* < 0.05) [41]. Furthermore, Hiraoka et al. assessed the repeatability of ocular biometric measurements using the IOL Master 500 (Carl Zeiss AG, Oberkochen, Germany) in cataract patients [42]. Their findings indicated that the repeatability of the steep meridian of corneal curvature radius, measured on the same day, was significantly worse in the DED group compared to controls, with values of 0.21 D vs. 0.14 D (*p* = 0.044) [42].

Roggla et al. reported significant keratometry changes within the first 5 min following the instilling of low- and high-viscosity lubricating eye drops [43]. In both patients, those with DED and healthy controls, there was a statistically significant difference in keratometry measurements after application of low- and high-viscosity eyedrops (*p* < 0.01). This difference was most pronounced at the 30-s mark and decreased at the 2- and 5-min intervals [43]. Notably, the percentage of patients experiencing a keratometry fluctuation > 0.5 D at 30 s was higher in the DED group: 34.3% with high-viscosity drops and 27.8% with low-viscosity drops, compared to 13.2% in the control group for both types of drops. These findings imply that keratometric measurements should be taken either without instilling eye drops or at least 5 min after instilling [43].

## 6. Approach to Dry Eye Disease in Patients Undergoing Cataract Surgery

DED can significantly impact the reliability and repeatability of preoperative ocular biometry measurements, potentially leading to IOL miscalculations, poor visual outcomes, and increased patient dissatisfaction. In the sections below, we will thoroughly discuss the preoperative, intraoperative, and postoperative considerations for patients with DED who are undergoing cataract surgery.

### 6.1. Preoperative Assessment of DED

A comprehensive clinical history is essential to identify any risk factors for DED. These factors include age, sex, smoking status, environmental and lifestyle influences, history of systemic diseases and medications, previous ocular procedures, and contact lens (CL) wear, among others [11,18]. DED validated questionnaires are crucial for detecting, grading, and assessing the quality of life of the patient. A short, practical, and self-applied survey instrument is preferred for this purpose and can be administered even before the patient is seen at the first or subsequent visit. The OSDI, the five-item Dry Eye Questionnaire (DEQ-5), and the Symptom Assessment iN Dry Eye (SANDE) are some of the most popular instruments used [44]. On the other hand, there are numerous invasive and non-invasive, qualitative and quantitative clinical tests available to evaluate patients with DED. However, some of these tests may lack adequate sensitivity and specificity to accurately detect and diagnose DED. Therefore, their use should be approached with caution to avoid lengthy procedures and unnecessary costs [4]. The order and sequence of the diagnostic tests is crucial to prevent contamination and ensure the most accurate measurements [45]. Non-invasive tests, such as interferometry, aberrometry, thermography, and tomography, among others, should be conducted first or on a subsequent visit. Invasive tests, such as vital staining, Schirmer’s test, and Meibomian gland functionality assessments, are to be performed only after the non-invasive tests. Lastly, the physico-chemical properties of tears, including osmolarity, lactoferrin, lysozyme, and matrix metalloproteinase (MMP)-9 levels should be measured during a different visit [45].

#### 6.1.1. DED Questionnaires

DED questionnaires are essential for standardization and characterization of the signs and symptoms associated with DED [46]. Although the OSDI is the most frequently used questionnaire, it has several drawbacks. It is time-consuming and does not include common dry eye symptoms such as foreign body sensation and tearing. Additionally, it addresses the impact of DED only on a limited range of daily activities, including reading, driving at night, computer use, and watching TV. Consequently, it does not fully capture the effects of DED on patients’ daily lives [47]. While TFOS DEWS II recommends using the OSDI and the DEQ-5 as part of the DED diagnostic algorithm. In contrast, the TFOS DEWS III suggests a shortened 6-item version of the OSDI [48,49]. However, none of these questionnaires were specifically designed for evaluating preoperative patients. The Standard Patient Evaluation of Eye Dryness (SPEED) is another dry eye questionnaire that assesses the frequency and severity of eye irritation and its impact on daily life [50]. In 2019, the American Society of Cataract and Refractive Surgery (ASCRS) developed the SPEED II preoperative questionnaire for ocular surface disease (OSD) evaluation in patients undergoing cornea- and lens-based refractive surgery. The SPEED II includes items aimed at screening the use of lubricating drops, fluctuating vision that improves with blinking and/or lubrication, and patient expectations [51]. Considering the limitations of questionnaires like OSDI and DEQ-5, ophthalmologists should consider the SPEED II Preop OSD Questionnaire for assessing and screening patients with DED who are undergoing cataract surgery.

#### 6.1.2. Ocular Surface Staining

**Figure 2:**
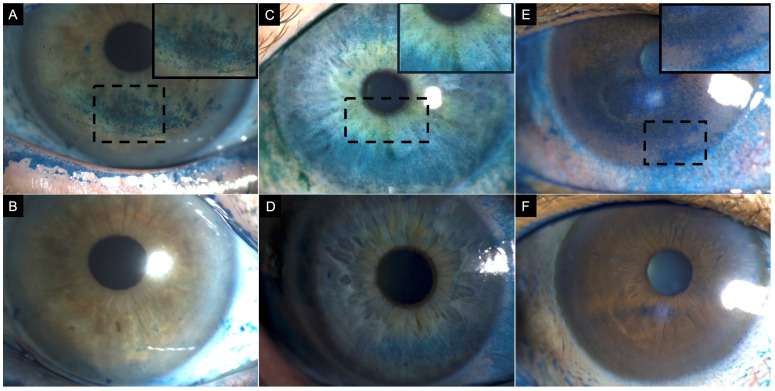
Clinical images of three patients with corneal lissamine green staining before (top row) and after treatment (bottom row). (**A**) A 77-year-old female patient with definite ocular graft-versus-host disease showing corneal lissamine green staining inferiorly (3 points), nasally (2 points), and temporally (2 points); total National Eye Institute (NEI) grading score of 7. (**B**) After treatment, staining was limited to the inferior region (1 point). (**C**) A 57-year-old female patient with Sjögren syndrome showing corneal lissamine green staining inferiorly (3 points), nasally (1 point), temporally (1 point), and superiorly (2 points) (2); with a total NEI grade score of 7. (**D**) After treatment, staining was limited to the inferior (2 points) and temporal (2 points) corneal quadrant. (**E**) A 60-year-old female patient with Sjögren syndrome showing corneal lissamine green staining centrally (3 points), inferiorly (3 points), nasally (3 points), temporally (2 points), and superiorly (2 points) with a total NEI grading score of 13. (**F**) After treatment, resolution of corneal staining, NEI grading score 0. Inset demonstrates a magnified view of the region with corneal lissamine green staining to enhance clarity. Note: While sodium fluorescein is the conventional standard for evaluating corneal epithelial damage, lissamine green is employed here to specifically visualize pre- and post-treatment differences in punctate epithelial staining.

**Figure 3:**
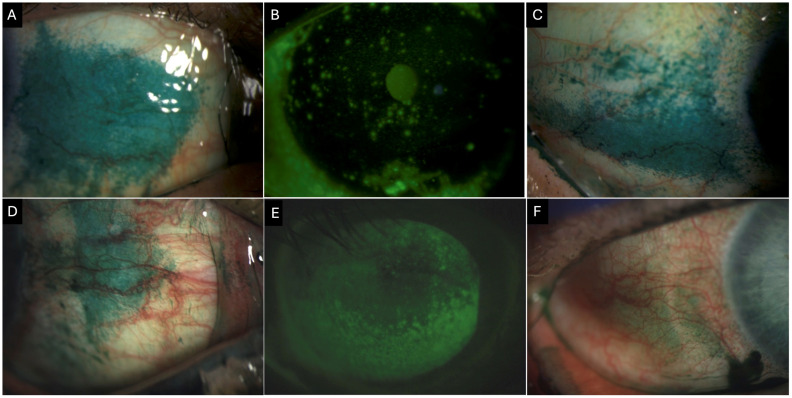
Clinical images of a 55-year-old female patient with Primary Sjogren’s syndrome showing (**A**) conjunctival lissamine green staining of the nasal quadrant (3 points). (**B**) Corneal fluorescein staining showing positive staining centrally (1 point), inferior (2 points), nasally (2 points), temporally (2 points), and superiorly (2 points) with a total National Eye Institute (NEI) grading score of 9. (**C**) Conjunctival lissamine green staining of the temporal quadrant (3 points) Clinical images of a 69-year-old female patient with definitive ocular graft-versus-host disease. (**D**) Conjunctival lissamine staining of the nasal quadrant (3 points). (**E**) Corneal fluorescence staining shows positive staining inferiorly (3 points), nasally (3 points), temporally (3 points), and superiorly (2 points), with a total NEI score of 11. (**F**) Conjunctival fluorescein staining of the temporal quadrant (2 points).

Ocular surface staining is a crucial component of dry eye assessment. Conjunctival and corneal staining are features of many disorders; however, the staining density and distribution may point towards a specific diagnosis [52]. The most frequently used dyes are fluorescein for the cornea and lissamine green for the conjunctiva [53]. While fluorescein penetrates through intercellular spaces of damaged epithelium, the latter vital dye stains devitalized epithelial cells and those devoid of mucin protection [52]. There are several classification systems used to assess the extent of epithelial damage on the ocular surface. Notable among these are the National Eye Institute (NEI) corneal fluorescein staining (CFS) score, the Oxford scheme, and the ocular surface staining (OSS) score developed by the Sjögren’s International Collaborative Clinical Alliance (SICCA) [54,55,56]. In patients with significant corneal central staining that are being evaluated for cataract surgery, particular attention is required to avoid performing keratometry and biometric analysis until the corneal surface is restored (Figure 2 and Figure 3).

#### 6.1.3. Lid Wiper Epitheliopathy (LWE)

Lid wiper epitheliopathy (LWE) is a frequently overlooked condition found in CL users and patients with DED [18]. The “lid wiper” region extends from the mucocutaneous junction (line of Marx) to the subtarsal fold superiorly, and from the lateral canthus to the medial upper punctum horizontally. Its function is to spread tears during blinking. Continuous staining of this area with lissamine green dye indicates the presence of LWE [57]. In a study involving 443 eyes of non-CL wearers, the prevalence of LWE was found to be 12.5% in the lower eyelid and 39.5% in the upper eyelid [58]. Furthermore, the prevalence of LWE was significantly higher among soft contact lens wearers (58.1% in the lower eyelid and 68.1% in the upper eyelid) and rigid gas permeable CL (RGP-CL) users with rates of 84.4% in the upper eyelid and 71.9% in the lower eyelid [58]. Another study by Korb et al. reported an 88% prevalence of LWE among symptomatic DED patients and only 16% among asymptomatic patients [59]. Although there have been no studies investigating the effect of LWE on ocular biometry measurements of patients undergoing cataract surgery, its presence may indicate significant ocular surface dryness and could assist in diagnosing symptomatic DED.

#### 6.1.4. Meibomian Gland Functionality

Meibomian gland functionality is an essential component of the preoperative evaluation of cataract surgery patients since it represents the leading cause of evaporative DED [60,61,62]. Both the anterior and posterior palpebral margins should be examined for signs of infection and inflammation. Increased thickness, rounding of the posterior square edge, notching, telangiectasia, madarosis, as well as orifice capping, retro-placement, and pouting [63]. Additionally, blinking insufficiency, anterior inflammation, and secretions, such as crusts, scales, collars, or sleeves, along with changes in the mucocutaneous junction, including anterior or posterior displacement, striation, keratinization, and mucosal absorption, should also be evaluated [60]. Cochener et al. reported that 52% of patients presenting for cataract surgery had MGD [64]. Detecting MGD, tear film instability, and evaporative DED primarily involves assessing the proportion of glands in each eyelid that can express meibum and the quality of the meibum released, which can vary from fluid and transparent to cloudy, cloudy with particles, or thick and pasty [63,65,66]. Additionally, non-invasive infrared meibography allows in vivo visualization of the morphological characteristics of the meibomian glands. It can be used to evaluate the response to treatment objectively [67]. Furthermore, numerous studies have reported that cataract surgery may alter meibomian gland morphology and function and worsen dry eye symptoms [68,69,70].

#### 6.1.5. Tear Film Breakup Time (TFBUT)

Tear film breakup time (TFBUT) is a critical and sensitive measure used in evaluating the health of the ocular surface. A shortened TFBUT often results from the thinning of the tear film due to hyposecretion of the accessory and main lacrimal glands. This thinning can occur alone or in conjunction with a reduction of the oily phase due to MGD. A short TFBUT leads to rapid evaporation and a shortening of the tear breakup time. Research indicates that TFBUT has excellent diagnostic accuracy, with an optimal cutoff point between 5.3 and 6.0 s for differentiating healthy patients from those with DED. The prospective health assessment of cataract patients’ ocular surface (PHACO) study found a high prevalence of DED among patients scheduled for cataract surgery, with 62.9% of them having a TFBUT ≤ 5 s [71]. Furthermore, a prospective study examining patients before and after cataract surgery revealed that 45.8% had a TFBUT shorter than normal prior to surgery, increasing to 54.2% one month after the procedure. On average, the TFBUT significantly decreased from 8.63 s to 7.73 s following cataract surgery (*p* = 0.004) [72]. Many studies have also documented a notable reduction in TFBUT among patients undergoing cataract surgery, emphasizing the importance of conducting this sensitive DED clinical test both before and after surgery [73].

#### 6.1.6. Corneal Sensitivity

**Figure 4:**
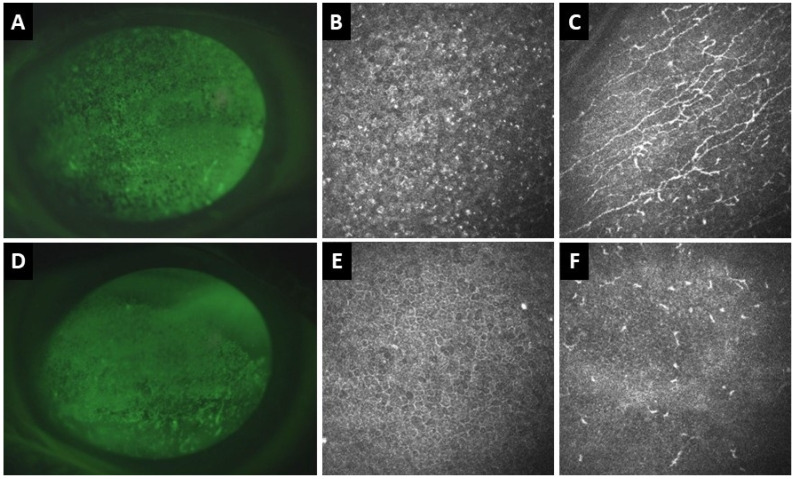
Clinical and in vivo confocal microscopy (IVCM) images of the right (**A**–**C**) and left (**D**–**F**) eyes of a 77-year-old patient with definite ocular graft-versus-host disease. (**A**) Corneal fluorescence staining of the right eye shows superficial punctate keratitis (SPK). (**B**) IVCM shows hyperreflective epithelial dots and (**C**) activated dendritic cells at the sub-basal nerve plexus with increased corneal nerve tortuosity. (**D**) In the left eye, there is also corneal SPK. (**E**) IVCM demonstrates corneal epithelial squamous metaplasia and (**F**) absence of corneal nerves and inflammatory cells at the sub-basal nerve plexus.

Corneal sensitivity serves as a surrogate marker for the health of the ocular surface. Corneal nerves regulate the blinking and tearing reflexes, transmit sensation, and modulate wound healing [74]. Corneal sensitivity can be assessed with a cotton wisp, the Cochet-Bonnet esthesiometer, and with the recently approved Brill non-contact esthesiometer [75,76]. Lyne reported that 29 of 29 eyes (100%) had almost complete anesthesia in the superior half of the cornea one year after extracapsular cataract extraction (ECCE), with only 3 (10%) recovering normal sensitivity two years after [77]. In otherwise healthy eyes, complete recovery of corneal sensitivity has been observed three months following phacoemulsification surgery [78]. However, in patients with pre-existing OSD, disruption of corneal nerves during cataract surgery may lead to increased epithelial permeability and reduced wound healing, which can be assessed with in-vivo confocal microscopy (Figure 4) [78,79].

#### 6.1.7. Tear Hyperosmolarity

Tear hyperosmolarity is a well-known surrogate marker of DED, and it is considered the most sensitive individual measure of this condition [80]. Normal values for tear osmolarity range from 308 to 316 mOsm/L [4]. Epitropoulos et al. conducted a study comparing the repeatability of keratometry measurements between hyperosmolar patients (>316 mOsm/L) and controls (<308 mOsm/L) who were undergoing cataract surgery. Measurements were taken within 3 weeks [81]. The percentage of eyes with a difference in the Km (8% vs. 0%, *p* = 0.049) and IOL power difference (10% vs. 0%, *p* = 0.02) of >0.5 D was significantly higher in the hyperosmolar group [81]. According to the recent preoperative diagnostic algorithm for OSD by the ASCRS Cornea Clinical Committee, tear osmolarity is recognized as an essential screening test [51].

#### 6.1.8. Matrix Metalloproteinase (MMP)-9

Matrix metalloproteinase (MMP)-9 is a proteolytic enzyme produced by stressed epithelial cells. It plays a role in a signal transduction cascade that leads to the induction of various pro-inflammatory cytokines, including interleukin (IL)-1, tumor necrosis factor (TNF)-alpha, and transforming growth factor (TGF)-beta, among others [82]. The sensitivity, specificity, negative predictive value, and positive predictive value of point-of-care MMP-9 testing for diagnosing inflammation related to DED are 85%, 94%, 73%, and 97%, respectively [83]. The significance of preoperative MMP-9 testing is two-fold. First, elevated levels of MMP-9 can delay corneal wound healing. In the context of cataract surgery, DED and the desiccating stress that occurs during the procedure stimulate the production of MMP-9 in ocular surface epithelia [82,84]. In a murine model, Pflugfelder et al. demonstrated that increased MMP-9 levels disrupt the corneal epithelial barrier function, likely by degrading tight junction proteins, such as occludins in superficial corneal epithelial cells [85]. Second, patients with elevated MMP-9 and/or tear osmolarity may benefit from preoperative treatment with anti-inflammatory eyedrops such as cyclosporine (CsA), lifitegrast, or a short course of topical corticosteroids (TCS) [51,86].

### 6.2. Preoperative Treatment of DED

According to TFOS DEWS II, the first step in treating DED is to modify, whenever possible, environmental triggers associated with the condition. These triggers may include various factors such as topical and systemic medications, dry conditions, environmental pollutants, excessive use of digital devices, and long-term contact lens wear. In addition to addressing these triggers, patients can benefit from treatments like artificial tears, warm compresses, eyelid hygiene, and nutritional supplements [5]. However, these therapies may not be sufficient for patients with moderate to severe DED. There is also a pressing need for rapid restoration of the tear film and ocular surface homeostasis to ensure accurate ocular biometry measurements and to enhance postoperative outcomes [51]. As a result, patients undergoing cataract surgery may require a more aggressive treatment approach. Because multiple inflammatory pathways are active on the ocular surface, a combinatorial approach may be more effective than the traditional step-up strategy for patients with recalcitrant OSD and severe dry eye subtypes, such as ocular GvHD [87,88].

In patients preparing for cataract surgery with preexisting DED, treatment should target the underlying pathogenic driver(s) of disease, rather than follow strict criteria for management based on a mild, moderate, or severe classification. The presence of MGD, for example, may require lipid-containing lubricants, slit-lamp meibomian gland expression, supplementation with omega-3 fatty acids, antibiotics, or thermal therapies [4,89]. DED driven by inflammatory mechanisms may benefit from the use of topical immunomodulatory drops such as CsA or Lifitegrast [4,89]. The TFOS Dews II Report emphasized that DED subtypes are not mutually exclusive, but rather a continuum. For example, elements of both MGD-driven and aqueous deficient dry eye may be present in a patient preparing for cataract surgery [5,18]. Therapeutic response to any treatment needs to be evaluated at every visit, including a reassessment of the pathogenic drivers [4,89]. The following subsections will summarize DED treatments, with a focus on the pathogenic factors they target and their impact on patients preparing for cataract surgery.

#### 6.2.1. Lubricant Eye Drops

Lubricant eye drops are the primary treatment of DED. They can be classified into two main categories, with the first being water-soluble polymers, such as cellulose and polyvinyl derivatives, polyethylene glycol, propylene glycol, and hyaluronic acid. These formulations enhance viscosity and improve water retention in the eye. The other type are lipid-based lubricants, which are aimed at restoring the lipid layer of the tear film to prevent its evaporation [89]. Another way to categorize lubricant eye drops is by their preservation status, into preserved and unpreserved formulations. While unpreserved formulations are generally preferred to minimize ocular surface toxicity—especially in patients who require frequent use and long-term treatment—certain compounds in pharmaceutical formulas may be unstable and at risk of contamination without a preservative agent. Eye care specialists must be aware of the physicochemical properties of each artificial tear formulation, and treatment decisions should depend on the subtype and severity of DED [5,89].

#### 6.2.2. Lid Margin Disease Therapy

Lid margin disease therapy focuses on managing blepharitis and MGD. Research indicates that symptoms of blepharitis, MGD, and dry eye may worsen following cataract surgery [90,91,92]. A randomized controlled trial (RCT) involving patients with MGD showed that performing eyelid hygiene twice a day from three days before to one week after cataract surgery significantly reduced postoperative dry eye symptoms, improved the expressibility and secretion of the meibomian glands, and prevented exacerbation of anterior blepharitis [92]. Intense pulsed light (IPL) therapy delivers continuous pulses of light with wavelengths ranging from 500–1200 nm. While it has been traditionally used by dermatologists to treat skin vascular lesions, Toyos et al. (2015) were the first to demonstrated its efficacy for MGD-associated DED [93]. Since then, multiple studies have indicated that the combination of IPL with manual meibomian gland expression (IPL-MGX) is more effective than either treatment alone [94,95]. In a recent study involving 67 consecutive patients with MGD-associated DED who were scheduled for cataract surgery with diffractive trifocal IOL implantation, Teshigawara et al. evaluated the efficacy of IPL-MGX on postoperative visual outcomes [96]. Four IPL-MGX sessions were conducted every two weeks preoperatively in one eye, while the fellow eye served as a control. The results showed significant improvements in the TFBUT, corneal SPK, and HOAs both preoperatively and at one week, one month, and three months after cataract surgery. Moreover, the group receiving IPL-MGX exhibited significantly better postoperative contrast sensitivity and visual acuity at each time point [96]. Kawagoe et al. reported that IPL-MGX also improved the accuracy of postoperative refraction in patients with MGD-associated DED undergoing cataract surgery [97]. Additionally, vectored thermal pulsation (VTP) is another Food and Drug Administration (FDA)-approved MGD treatment that combines massage with heat therapy. Preoperative VTP has also been shown to improve meibomian gland function, corneal SPK, TFBUT scores, and alleviate dry eye symptoms after cataract surgery [98,99,100,101,102].

#### 6.2.3. Anti-Inflammatory Alternatives

Anti-inflammatory alternatives are part of the second step in the management algorithm for DED outlined in the TFOS DEWS II Report. These alternatives should be considered in specific situations, particularly for patients with elevated levels of MMP-9 [5]. Even in cases of moderate DED, there is often a subclinical inflammatory response in the lacrimal gland and ocular surface that requires anti-inflammatory treatment. A short course of TCS, CsA, and lifitegrast are commonly used as anti-inflammatory agents in managing DED.

##### Topical Corticosteroids (TCS)

Topical corticosteroids (TCS) effectively reduce pro-inflammatory mediators, such as adhesion molecules, cytokines, and MMPs, which contribute to ocular surface inflammation [103]. However, their known side effects limit their long-term use. Loteprednol etabonate (LE), a topical steroid derived from an inactive metabolite of prednisolone acetate is designed to minimize side effects. Upon administration, LE metabolizes rapidly, reducing the risk of excessive exposure [104]. A double-masked RCT found that 0.5% LE twice daily for two weeks, followed by once daily for two weeks, and twice weekly for four weeks, resulted in significant improvement in symptom severity on day 28. It also reduced the expression of human leukocyte antigen (HLA)-DR expression in CD45+ conjunctival cells on day 14 compared to a control group treated with 0.9% sodium chloride. Importantly, no adverse effects were reported in either group [105]. Despite the reported safety and efficacy of TCS in managing DED, a recent Cochrane meta-analysis indicated that the quality of the evidence is of moderate to low certainty due to high risk of bias related to selective results reporting [106]. We recommend a short course of CTS lasting 2 to 4 weeks for patients undergoing cataract surgery that requires anti-inflammatory management.

##### Cyclosporine A (CsA)

Cyclosporine A (CsA) is a calcineurin inhibitor that the FDA has approved as a 0.05% emulsion, for managing DED. Donnenfeld et al. reported that administering 0.05% CsA twice daily, starting one before and continuing for two months after cataract surgery, resulted in significant improvements in postoperative visual acuity, corneal and conjunctival staining, TFBUT scores, and contrast sensitivity compared with artificial tears in patients who underwent cataract surgery with multifocal IOL implantation [107]. A new water-free formulation of 0.1% CsA also demonstrated a statistically significant improvement in total and central CFS in DED patients prior to cataract surgery [108]. An RCT showed that induction therapy with 0.5% LE administered four times daily for two weeks, followed by 0.05% CsA twice daily for six weeks, led to a significant reduction in the stinging associated with CsA, as well as improved Schirmer scores, and ocular surface staining compared to CsA and artificial tears alone [109].

##### Lifitegrast

Lifitegrast is an FDA-approved lymphocyte function-associated antigen-1 antagonist that reduces ocular surface inflammation by blocking T cell binding to intercellular adhesion molecule (ICAM)-1 [110]. The effectiveness of lifitegrast in managing DED has been demonstrated in multiple RCTs [111,112,113]. Hovanesian et al. reported that the accuracy of biometry measurements for the achieved refractive spherical equivalent was within 0.25 D (47% and 50%), 0.50 D (71% and 79%), and 0.75 D (81% and 91%) before and after treatment with lifitegrast in eyes with DED undergoing cataract surgery [114]. Additionally, improvements in SPEED scores, CFS, TFBUT, and conjunctival hyperemia were observed after treatment with lifitegrast [114].

##### Lactoferrin

Lactoferrin is a glycoprotein found in tears, and it serves multiple functions such as anti-inflammatory and antimicrobial properties. It also promotes cell growth, inhibits angiogenesis, and exhibits antitumoral effects [115]. Current research indicates a link between low levels of lactoferrin in tears and various forms of dry eye, including primary and secondary Sjögren syndrome, as well as dry eye not associated with Sjögren [116]. Additionally, oral lactoferrin has demonstrated positive results as a treatment for both Sjögren’s and non-Sjögren’s dry eyes. However, there is limited research focusing on the use of oral lactoferrin in the treatment of DED following surgery. A non-blinded, prospective randomized controlled trial with a concurrent parallel design evaluated the dry eyes induced by manual small incision cataract surgery, and the effect if any, of oral lactoferrin on those suffering from the condition [115]. Two groups were created, an experimental group received oral lactoferrin 350 mg/day from day one after cataract surgery. The control group did not receive lactoferrin, and both groups received the routine postoperative medications. The mean TFBUT showed a significantly improvement in ethe lactoferrin group compared to controls (*p* < 0.001). Additionally, at 60 days postoperative, the lactoferrin-treated group showed a progressive increase in Schirmer test values compared to controls (*p* = 0.0001) [115]. Recently, a liposomal-lactoferrin-based eye drop formulation has demonstrated a promising role for lactoferrin as a safe and targeted treatment. It aims to enhance the physiological defenses of the ocular surface and control bacterial contamination before cataract surgery [117]. This research opens up new possibilities for exploring the use of topical lactoferrin supplementation in managing dry eye and potential infections related to anterior segment surgeries, including cataract extraction.

#### 6.2.4. Autologous Serum (AST)

**Figure 5:**
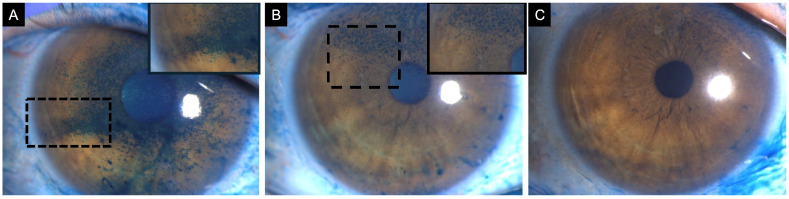
A 47-year-old female patient diagnosed with definite ocular-graft-versus-host disease scheduled for cataract surgery. (**A**) Baseline corneal lissamine green staining showing positive staining centrally (3 points), inferior (2 points), temporally (2 points), nasally (3 points), and superiorly (3 points), with a total National Eye Institute (NEI) grading score of 13. The patient started treatment with topical 1% methylprednisolone, 5% pooled human immunoglobulin, 50% autologous serum tears, artificial tears, and contact lenses. (**B**) After 2 months of therapy, corneal lissamine green staining was reduced, and limited only to the superior quadrant (3 points). (**C**) Post-cataract surgery, corneal lissamine green staining was present only in the superior quadrant (1 point). Insets demonstrate a magnified view of the region with corneal lissamine green staining to enhance clarity.

Autologous serum (AST) possesses biochemical properties, including PH, nutrients, vitamins, fibronectin, and growth factors, that closely resemble those of the natural tears [5]. As a result, AST is an important treatment option for patients with moderate to severe DED, to help restore the ocular surface and promote healing. Lei et al. reported that in patients developing dry eye following cataract surgery, combinatorial therapy including AST and sodium hyaluronate resulted in significantly better improvement in both signs and symptoms of dry eye disease compared to treatment with only sodium hyaluronate [118]. These patients were also on topical corticosteroids in addition to autologous serum tears. Our group has also found similar results in GvHD patients undergoing cataract surgery (Figure 5). Combinatorial therapy, including AST and anti-inflammatory therapies, reduced complications like corneal melts, epithelial defects, etc., in patients with GvHD-related DED compared to the incidence reported in the literature [37]. A study evaluating patients with recalcitrant DED reported a median response time of two weeks in nearly 80% of participants, highlighting the potential of AST to rapidly stabilize the ocular surface and making it beneficial for patients awaiting cataract surgery [119]. Despite these benefits, AST use is logistically challenging as well as variability in treatment response has been reported. However, clinicians specializing in OSD widely regard AST as a critical therapeutic option of the OSD management armamentarium [120].

#### 6.2.5. Contact Lenses (CLs)

Contact lenses (CLs) play an important role in the management of dry eye disease patients by protecting the ocular surface from environmental insults, mechanical damage from abnormal lids, and keeping the ocular surface moist. CLs have emerged as an effective adjunct in managing dry eye disease in the perioperative cataract settings as well. In a prospective study by Wu et al. including 120 patients with DED undergoing cataract surgery, patients fitted with bandage CLs (BCLs) showed greater improvement in tear stability at one month compared to controls. Notably, postoperative increases in tear inflammatory cytokines (IL-6, IL-8, ICAM-1) observed in controls were absent in the BCL group [121]. Similar findings were reported by Chen et al. who reported reduced ocular symptoms and fluorescein staining scores in BCL-treated eyes after complicated cataract or IOL surgeries [122]. For patients who are habitual contact lens wearers, preoperative discontinuation is essential to ensure accurate biometry and IOL power calculation. Continued wear, particularly of rigid gas-permeable or scleral lenses, can transiently alter corneal curvature and induce refractive changes [123]. To ensure accurate corneal measurements, soft CLs are ideally discontinued for at least 1 week before surgery [37]. These findings support the role of CLs as a valuable therapeutic strategy for optimizing the ocular surface in cataract patients with DED.

## 7. Intraoperative Considerations in Dry Eye Disease

**Figure 6:**
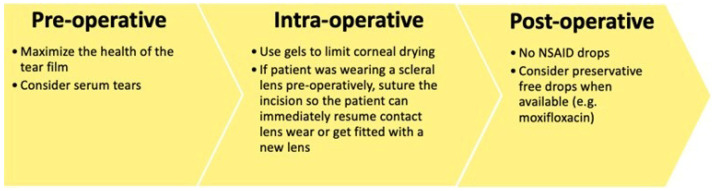
Special pre-, intra-, and post-operative considerations in patients with severe dry eye disease.

Recent advances in cataract surgery over the past few decades have made it a safe, minimally invasive procedure, allowing for quick visual recovery [124]. However, even the most advanced techniques still pose potential risks to the ocular surface. These risks include corneal nerve damage and reduced sensitivity induced by clear corneal incisions, toxicity from topical medications, and the effect of repeated surface drying and irrigation [9,125,126]. Such injuries can disrupt tear film stability, potentially leading to the onset of DED [9,127]. Below is an overview of the intraoperative factors that may mitigate these effects (Figure 6).

### 7.1. Corneal Nerve Damage and Surgical Technique

Corneal nerves play a key role in ocular surface homeostasis [74]. The corneal incision performed during cataract surgery can transect the subbasal nerve plexus, resulting in reduced corneal sensitivity [77,125,128]. In large incision ECCE, where a full-thickness incision approximately 12 mm is made, sensitivity may take more than 2 years to return to preoperative levels [77]. John evaluated corneal sensation after a 5 mm scleral tunnel incision and reported a decrease in corneal sensitivity across the incision width, extending in a wedge-shaped pattern, with incomplete recovery after one month [128]. Improvements in cataract surgery have enabled progressively smaller incisions. Nevertheless, even more modern, smaller, clear corneal incisions may disrupt corneal sensitivity. In a study of 50 eyes, Kim et al. observed a decrease in corneal sensation at the incision site after a 3 mm clear corneal phacoemulsification incision [128]. This reduction was more pronounced in temporal than in superior incisions; however, in both groups, sensitivity returned to baseline levels by 3 months. However, subbasal nerve density measured by confocal microscopy did not completely recover to the preoperative level by 3 months. This finding suggests that morphological recovery might take longer than recovery of corneal sensitivity [128].

While modern phacoemulsification techniques have minimized incision sizes, new technologies like femtosecond laser-assisted cataract surgery (FLACS) introduce additional factors that may impact corneal innervation. Both FLACS and manual cataract surgery (MCS) can potentially cause ocular damage, including nerves injury as previously discussed. However, FLACS may present an extra risk for ocular surface injury, primarily due to the use of the patient interface [129]. Research indicates that patients undergoing FLACS exhibit a greater release of pro-inflammatory markers in aqueous humor compared to those undergoing standard MCS [130,131]. Furthermore, recent evidence suggests that elevated levels of cytokines may persist for several years after cataract surgery, potentially leading to a chronic subclinical inflammatory state [132]. Other contributing factors may include longer surgical time, damage to conjunctival goblet cells, and injury to ocular surface nerves from the vacuum and compression of the patient interface [129]. In an RCT, Shao et al. found that patients in the FLACS group experienced worse dry eye symptom scores and greater CFS during the initial postoperative period compared to those in the FLACS group [133]. However, by three months post-surgery, there were no significant differences between groups. These findings were further supported by a meta-analysis of six studies, which showed that FLACS was linked to notably worse dry eye symptoms and increased ocular surface staining in the early postoperative phase, although these differences di not persist beyond three months [134].

### 7.2. Ocular Surface Desiccation and Phototoxicity

The healthy corneal surface is naturally moist and smooth, serving as the primary refractive surface of the eye. However, during cataract surgery, the cornea is frequently exposed to air and irrigated with balanced saline solution (BSS), which can disrupt the stability of the tear film and cause desiccating stress. These events may contribute to DED related to cataract surgery [9]. Studies have shown that using intraoperative viscoelastic agents on the cornea instead of BSS can reduce symptoms and metrics of DED [135,136,137]. For instance, an RCT involving 149 patients compared the intraoperative application of 2% hydroxypropyl methylcellulose (HPMC) to BSS for coating the ocular surface during cataract surgery. The results indicated that HPMC improved tear production, as measured by Schirmer I test, and reduced ocular staining after surgery, particularly in patients with preexisting DED [136]. Additionally, Yusufu et al. conducted a study that compared HPMC corneal surface coating to BSS during phacoemulsification. Their findings revealed significantly better postoperative outcomes in Schirmer I measurements, TFBUT, and CFS in elderly and diabetic patients treated with HPMC [135]. Furthermore, using tripolymeric coating gel, which contains HPMC, xanthan gum, and carrageenan, during cataract surgery led to better preservation of the corneal epithelium and improved surface recovery compared to irrigation of BSS alone. This finding was supported by anterior segment OCT and in-vivo confocal microscopy [137].

Continuous illumination from the surgical microscope can contribute to intraoperative stress on the ocular surface, potentially leading to phototoxic damage [9,138]. In a prospective study by Cho et al., longer exposure times to the microscope light during cataract surgery were associated with shorter tear break-up time and increased ocular discomfort in DED patients [138]. Additionally, Hwang et al. demonstrated in a rabbit model that exposure to operating microscope light resulted in reduced tear secretion, goblet cell loss, ultrastructural damage to corneal cells and conjunctival tissues, and increased expression of inflammatory cytokines. These changes on the ocular surface are consistent with DED, and the effects tend to be more pronounced at higher light intensities [18,139]. While the specific impact of microscope light phototoxicity is not fully understood, minimizing intraoperative exposure may help preserve ocular surface integrity, especially in patients with preexisting DED.

### 7.3. Ocular Surface Antisepsis

During cataract surgery, the ocular surface is frequently exposed to a variety of pharmacologic agents. While these agents are essential for preventing infection, providing anesthesia, and controlling inflammation, many can have direct or cumulative toxic effects on epithelial cells and tear film components [140,141]. Benzalkonium chloride (BAK) is the most commonly used preservative, found in approximately 70% of ophthalmic formulations [140]. Although BAK enhances the corneal penetration of pharmacologic agents, it also destabilizes the tear film, induces epithelial apoptosis via oxidative stress and mitochondrial damage, and promotes inflammation. These effects can be harmful for eyes with preexisting DED [140].

Attention should also be given to the effects of povidone-iodine (PI), which may cause damage to ocular surface. Preoperative antisepsis using a 5–10% solution of PI applied to the ocular surface and periocular skin is regarded as the most effective strategy for preventing endophthalmitis. Its consistent application has been identified as the single most impactful measure in reducing infection rates over decades of surgical practice [142]. However, PI has been shown to adversely affect various ocular surface cell types, potentially impairing mucin production and delaying recovery of the ocular surface [143]. In an in vitro study conducted by Swift et al. exposure to PI demonstrated a negative impact on cell viability and metabolism in human conjunctival epithelial cells, goblet cells, and fibroblasts [143]. These findings suggest that while standard antisepsis protocols are necessary, they may also exacerbate ocular surface damage. To reduce the toxicity associated with PI, concentrations lower than 5% have been investigated in both laboratory and clinical settings [144,145]. In vitro results by Zwicker et al. indicated that 1.25% concentration of PI was as effective as the standard 5% concentration in reducing bacterial and fungal loads [144]. Consistent with these findings, a trial demonstrated that a 3-day course of topical 0.6% PI before cataract surgery achieved significant bacterial load reduction. The same trial showed that a 0.02% chlorhexidine solution, under identical conditions, proved to be more effective in reducing microbial load, and had better patient tolerability [145]. While lower concentrations of PI have demonstrated effectiveness in reducing bacterial load with less ocular surface toxicity, further studies are necessary to determine the optimal concentration and exposure time.

## 8. Postoperative Management of Dry Eye Disease After Cataract Surgery

Adequate postoperative management is necessary to prevent the detrimental effects of cataract surgery on the ocular surface [68,69,70]. Han et al. reported significantly worse ocular symptom scores, lid margin abnormalities, meibum expressibility, and TFBUT scores 3 months after cataract surgery [68]. Fujimoto et al. reported a 49% prevalence (21 of 43 patients) of dry eye symptoms 1 month after cataract surgery [69]. Female sex and upper eyelid meibomian gland loss area were deemed as statistically significant risk factors in multivariable analysis [69]. Thus, patients should be screened for DED within the first month after cataract surgery.

### 8.1. A Personalized Approach Based on Dry Eye Phenotype

The following subsections detail a phenotype-specific approach to management of the most common DED subtypes postoperatively with emphasis on targeting the pathogenic driver(s) of disease. Recommended postoperative tests to determine pathogenic driver(s) include MGD grade, TFBUT, fluorescein staining, Schirmer’s Test, MMP-9 level, the SPEED II Questionnaire, and esthesiometry [49]. Similarly to the preoperative assessment, these phenotypes are not mutually exclusive and testing to evaluate the presence of new or different pathogenic drivers should be conducted at each postoperative visit.

#### 8.1.1. Postoperative Evaporative DED (MGD-Driven)

Hallmarks in the postoperative diagnosis of the evaporative or MGD-driven presentation of DED include reduced meibum quality and expressibility and some scattered lid margin changes with or without ocular discomfort or photophobia [146]. These are the pathologic changes that contribute to a patient’s MGD grade. Following the International Workshop on Meibomian Gland Dysfunction algorithm, an MGD grade ≥ 2 represents the cutoff at which symptoms appear [146]. Increased intake of omega-3 fatty acids, eyelid hygeine, and manual meibomian gland expression are targeted therapeutic approaches to management of this DED phenotype. Additionally, anti-inflammatory topicals, oral tetracycline medications, and/or topical azithromycin may be added when earlier conservative treatment fails [146]. Thermal pulsation is a treatment for advanced MGD-related dry eye and has shown improved meibum secretion scores and TFBUTs after cataract surgery, however, there have been no statistically significant results showing improved OSDI scores or lipid layer thickness after cataract surgery [147]. More high-quality research is needed to confirm whether thermal pulsation is effective in this subtype of DED after cataract surgery.

#### 8.1.2. Postoperative Aqueous-Deficient and/or Inflammatory DED

Patients presenting with aqueous-deficient DED postoperatively may have faster TFBUTs and Schirmer’s Test scores < 5 mm. TFOS DEWS III emphasizes that tear supplements remain the cornerstone for aqueous-deficicent dry eye treatment and emphasizes the various formulations and combination drops gaining popularity that target specific mediators of DED [13]. If a patient presents with increased MMP-9 levels in addition to fast TFBUTs and Schirmer’s Test scores < 5 mm, topical drops containing antiinflammatory agents such as trehalose may be more beneficial. Perumal et al. found that a topical combination solution of 3% trehalose and 0.15% hyaluronic acid significantly improved OSDI scores at day 28, and TFBUT at day 56 [148]. In cases of severe aqueous-deficient dry eye where underlying autoimmune disease is present, other anti-inflammatory topicals such as cyclosporine A or lifetigrast may be warranted in addition to standard strategies for increasing tear quantity such as punctal plugs with preservative-free tears.

#### 8.1.3. Postoperative Neuropathic Pain in DED

The hallmark of neuropathic pain as a subtype of DED is a discordance of signs and symptoms. Generally, patients with this subtype report significantly more symptoms than observable signs upon clinical examination. Corneal staining and TFBUT may be unremarkable, but scores from symptom questionnaires such as SPEED II may report findings [149]. Targeted therapies for this subtype of DED include systemic neuropathic agents such as gabapentin, which targets the voltage-sensitive Ca^2+^ channels to regulate excitatory neurotransmitter release and reduce the hypersensitivity of corneal nerves contributing to symptoms [149]. Ongun et al. reported improved OSDI scores, TFBUT, and Schirmer’s Test results in patients with DED driven by neuropathic pain and ocular surface disease who were treated with a combination of gabapentin, artificial tears, and cyclosporine drops for at least 6 weeks [150]. As with any systemic medication, however, side effects are of important consideration and drowsiness, dizziness, depression and headache are frequently reported by patients taking gabapentin [149]. Other studies show similar efficacy of gabapentin as a therapeutic targeting the neuropathic DED symptoms when used in combination with other therapies that target the ocular surface and inflammation. In patients without evidence of ocular surface disease, however, ocular neuropathic pain may be better managed with NSAIDs [151,152].

#### 8.1.4. Postoperative DED Driven by Neurotrophic Keratitis

Neurotrophic keratitis represents a unique phenotype of DED with hallmarks of epithelial defects and decreased or total loss of corneal sensation [153]. A personalized approach to DED management for patients with this phenotype is complicated by the diagnostic process, which requires application of a physical stimulus to the corneal surface. Cochet-Bonnet aesthesiometry remains the gold standard for diagosis of neurotrophic keratitis with values < 40 mm indicative of reduced sensation [154]. It is important to consider that in a setting with suspected corneal damage, the use of an instrument that poses risk of corneal damage is of concern. Further, several therapeutic recommendations for the management of neurotrophic keratopathy overlap with those of other DED phenotypes, with the exception of amniotic membrane transplant (AMT) and human nerve growth factor. Thus, it is recommended to perform aesthesiometry after failure of other therapeutics such as preservative-free topical lubricants, bandage contact lenses, and avoidance of NSAIDs [154]. If aesthesiometry results demonstrate decreased corneal sensation and a diagnosis of neurotrophic keratitis warranting advanced therapy is made, specific treatments such as human nerve growth factor and AMT may then be necessary [155,156].

Recombinant human nerve growth factor (rh-NGF) is an approved treatment for neurotrophic keratitis with Pflugfelder et al. demonstrating higher healing rates and reductions in lesion size in a randomized double-masked study of 48 patients [155]. Cryopreserved amniotic membrane is another treatment option that protects the epithelial surface and may restore homeostasis by reducing inflammation, reducing scarring, and promoting regeneration of corneal nerves. AMT has demonstrated an increase in subbasal nerve density and reduction in pain severity in 8–14 months for patients with a placement duration average of 7 ± 3 days. This may be of special utility in patients with risk factors for this specific subtype of DED such as diabetes mellitus, radiation, herpes zoster ophthalmicus, or herpes simplex keratitis [155].

## 9. Other Treatments for Postoperative DED Independent of Phenotype

### 9.1. Preservative-Free (PF) Artificial Tears

A 2022 systematic review and meta-analysis reported a 37.4% prevalence of de-novo DED after cataract surgery. DED severity peaked at 1-day postoperative and persisted for 1–12 months [7]. Apart from the pre- and intra-operative considerations discussed above, there are several considerations for managing DED after cataract surgery. In an RCT, Jee et al. investigated the postoperative use of PF versus preserved sodium hyaluronate and fluorometholone eye drops in patients with preexisting DED undergoing cataract surgery. They found that the PF formulations resulted in better OSDI, TFBUT, Schirmer 1, and fluorescein staining scores, as well as impression cytology findings, and goblet cell counts two months after surgery [141]. Given these findings, using PF eyedrops is safer for maintaining ocular surface stability in patients with DED.

### 9.2. Non-Steroidal Anti-Inflammatory Drugs (NSAIDs)

Topical NSAIDs are widely used after cataract surgery as they have shown to decrease postoperative pain, inflammation, and the incidence of cystoid macular edema. However, their use is associated with SPK, corneal infiltrates, epithelial defects, and stromal melting [151,152]. These adverse effects are more common in patients with pre-existing OSD. Thus, topical NSAIDs should be restricted–or avoided–in high risk patients [151]. Kato et al. demonstrated that post-cataract topical diclofenac significantly decreased the conjunctival goblet cell density compared with baseline (86.5 ± 76.7 vs. 257.0 ± 188.7 cells/mm^2^, *p* = 0.002) [157]. Interestingly, the change in goblet cell density was not significantly different after cataract surgery in eyes treated with diclofenac combined with topical rebamipide [157]. The latter is a quinolone derivative originally used to prevent the adverse effects of oral NSAIDs in the gastrointestinal mucosa.

### 9.3. Mucin Secretagogues

Several studies have demonstrated the efficacy of secretagogues for DED after cataract surgery. Diquafasol is a secretagogue that activates the P2Y2 receptor, promoting the secretion of mucin by goblet cells and tears, thereby improving DED. A prospective RCT compared the therapeutic efficacy and safety of 0.3% diquafosol with 0.1% sodium hyaluronate during the postoperative period following cataract surgery [158]. The diquafasol group had significantly better TFBUT scores (*p* < 0.001), conjunctival staining scores (*p* = 0.001), and corneal staining scores (*p* = 0.045) compared with the sodium hyaluronate group [158]. In another RCT, Jun et al. found that treatment with PF 3% diquafasol was superior to preserved diquafasol and PF sodium hyaluronate in treating post-cataract DED [159]. Another study made a post hoc analysis on the effects of 3% diquafosol on HOAs in patients diagnosed with dry eye after cataract surgery [160]. The corneal aberrations changed from an upward curve to an almost constant value (a stable pattern) in the diquafosol group, but not in the artificial tears group. Also, the HOA fluctuation and stability indexes were significantly lower in the diquafosol group than in the artificial tears group (both, *p* = 0.004). The authors concluded that 0.3% diquafosol is effective to treat DED after cataract surgery with improvement in visual function [160].

## 10. Conclusions

DED is a frequently encountered ocular surface disorder in patients undergoing cataract surgery. If left untreated, DED may result in poor postoperative outcomes, increased risk of complications, and decreased patient satisfaction and QoL. Thus, a thorough preoperative examination assessing ocular surface health is necessary prior to cataract surgery. The latter may allow identifying subtle signs in asymptomatic patients. On the other hand, special considerations are necessary to accurately calculate IOL power and improve refractive and visual outcomes in patients with pre-existing DED. Intraoperative considerations include minimizing the exposure to the microscope light and the thermal energy delivered by the phacoemulsification device, avoiding large incisions, and using the remaining viscoelastic on the cornea at the end of surgery. Despite an uncomplicated and well-planned cataract surgery, development or worsening of DED remains common. Postoperative treatment with PF eyedrops and restricting the use of topical NSAIDs are necessary to improve dry eye symptoms regardless of DED subtype. Additional therapeutic strategies aimed at targeting a patient’s specific pathogenic drivers of disease represent a transformative shift to personalized medicine in postoperative care of patients undergoing cataract surgery.

## Data Availability

No new data were created or analyzed in this study. Data sharing is not applicable to this article.

## Abbreviations

The following abbreviations are used in this manuscript:

ASCRS	American Society of Cataract and Refractive Surgery
AST	Autologous serum tears
BAK	Benzalkonium chloride
BCL	Bandage contact lens
CFS	Corneal fluorescein staining
CL	Contact lens
CsA	Cyclosporine
D	Diopters
DED	Dry eye disease
DEQ-5	Dry Eye Questionnaire 5
DEWS	Dry Eye Workshop
ECCE	Extracapsular cataract extraction
FDA	Food and Drug Administration
FLACS	Femtosecond laser-assisted cataract surgery
GVHD	Graft-versus-host disease
HLA	Human leukocyte antigen
HOAs	High-order aberrations
HPMC	hydroxypropyl methylcellulose
HSCT	hematopoietic stem cell transplantation
ICAM-1	Intercellular adhesion molecule 1
IL	Interleukin
IOL	Intraocular lens
IPL	Intense pulsed light
IPL-MGX	Intense pulsed light with manual meibomian gland expression
IVCM	In vivo confocal microscopy
Km	Mean keratometry
LE	Loteprednol etabonate
LFA-1	Lymphocyte function-associated antigen-1
LWE	Lid wiper epitheliopathy
MCS	Manual cataract surgery
MGD	Meibomian gland dysfunction
MMP-9	Matrix metalloproteinase 9
MSVI	Moderate to severe visual impairment
NEI	National Eye Institute
NSAIDs	Non-steroidal anti-inflammatory drugs
OCT	Optical coherence tomography
OSD	Ocular surface disease
OSDI	Ocular Surface Disease Index
OSS	Ocular surface staining
PF	Preservative free
PI	Povidone iodine
PRGF	Plasma rich in growth factors
PROSE	Prosthetic Replacement of the Ocular Surface Ecosystem
PRP	Platelet-rich plasma
QoL	Quality of life
QoV	Quality of vision
rh-NGF	Recombinant human nerve growth factor
RCT	Randomized controlled trial
RGP	Rigid gas permeable
SANDE	Symptom Assessment in Dry Eye
SICCA	Sjögren’s International Collaborative Clinical Alliance
SOD	Superoxide dismutase
SPEED	Standard Patient Evaluation of Eye Dryness
SPK	Superficial punctate keratopathy
SS	Sjögren syndrome
TCS	Topical corticosteroids
TFBUT	Tear film breakup time
TFOS	Tear Film & Ocular Surface Society
TGF	Transforming growth factor
TNF	Tumor necrosis factor
VTP	Vectored thermal pulsation
